# Spatial Language Processing in the Blind: Evidence for a Supramodal Representation and Cortical Reorganization

**DOI:** 10.1371/journal.pone.0024253

**Published:** 2011-09-14

**Authors:** Marijn E. Struiksma, Matthijs L. Noordzij, Sebastiaan F. W. Neggers, Wendy M. Bosker, Albert Postma

**Affiliations:** 1 Experimental Psychology and Helmholtz Institute, Utrecht University, Utrecht, The Netherlands; 2 Utrecht Institute of Linguistics OTS, Utrecht University, Utrecht, The Netherlands; 3 Department of Cognitive Psychology and Ergonomics, University of Twente, Enschede, The Netherlands; 4 Division of Brain Research, Department of Psychiatry, Utrecht University Medical Centre, Utrecht, The Netherlands; 5 Faculty of Psychology and Neuroscience, Neuropsychology & Psychopharmacology, Maastricht University, Maastricht, The Netherlands; 6 Department of Neurology, University Medical Centre Utrecht, Utrecht, The Netherlands; The University of Plymouth, United Kingdom

## Abstract

Neuropsychological and imaging studies have shown that the left supramarginal gyrus (SMG) is specifically involved in processing spatial terms (e.g. *above, left of*), which locate places and objects in the world. The current fMRI study focused on the nature and specificity of representing spatial language in the left SMG by combining behavioral and neuronal activation data in blind and sighted individuals. Data from the blind provide an elegant way to test the supramodal representation hypothesis, i.e. abstract codes representing spatial relations yielding no activation differences between blind and sighted. Indeed, the left SMG was activated during spatial language processing in both blind and sighted individuals implying a supramodal representation of spatial and other dimensional relations which does not require visual experience to develop. However, in the absence of vision functional reorganization of the visual cortex is known to take place. An important consideration with respect to our finding is the amount of functional reorganization during language processing in our blind participants. Therefore, the participants also performed a verb generation task. We observed that only in the blind occipital areas were activated during covert language generation. Additionally, in the first task there was functional reorganization observed for processing language with a high linguistic load. As the visual cortex was not specifically active for spatial contents in the first task, and no reorganization was observed in the SMG, the latter finding further supports the notion that the left SMG is the main node for a supramodal representation of verbal spatial relations.

## Introduction

Spatial language is used to locate places and objects in the world. In the human the left supramarginal gyrus (SMG) brain in particular has been found to be crucial for processing spatial language. Neuropsychological patients with lesions to the left SMG show remarkable and specific difficulties in producing and understanding spatial terms, in particular locative prepositions such as *above* and *to the left of* [1]–[3]. Furthermore, a recent fMRI study by Noordzij et al. [4] revealed higher activity in the left SMG for spatial sentences, containing locative prepositions, than for non-spatial sentences (see also [5], [6]). This difference in activity was present in both a verbal and a visual-spatial context. The results by Noordzij et al. [4] could be explained by a supramodal representation of spatial information, implying that activity in this region exceeds information from the stimulus modality yielding similar activation for verbal and visual-spatial contexts [7]–[11] related to the spatial characteristics of the stimuli. Furthermore, a supramodal representation maintains a link with the input modality, which can explain behavioral differences between different input modalities. Noordzij et al. showed that participants responded faster when a spatial sentence was followed by a picture than when it was followed by a sentence. The authors argued that participants relied on an imagery strategy in the picture condition, instead of always using a propositional representation. This finding could be supported by a supramodal representation, which allows for flexible comparison between verbal and visual-spatial input. An alternative explanation could be that the left SMG activation is related to the input format rather than the representational format, i.e. the perceptual and verbal input that is presented visually. This would be in line with a multimodal representation which is linked to the perceived modality, in this case visual, and established in modality-specific brain areas [7], [11]. Visual and spatial imagery is often confounded, which is not surprising given that the visual modality has the highest spatial resolution [12], [13]. However, we consider visual imagery and spatial imagery to be separate processes. Visual features such as color, and brightness are represented by visual imagery. On the other hand, spatial imagery refers to spatial relationships between objects or parts of an object [14]. Moreover, shape information can be deduced from visual information, however, shape information is invariant, i.e. it no longer depends on the observer's point of view [15]. This dissociation between visual and spatial imagery has been supported by evidence from neuropsychological patients [16].

The aim of the present fMRI study was to further examine the nature of the spatial language representation. Blind individuals who have never had any visual experience provide a particularly relevant group of subjects. Without any visual experience it is not possible to use visual information to represent a spatial description. However, information from other input modalities could be used to generate a spatial mental representation. Furthermore, converging evidence has shown that blind individuals are able to generate a spatial mental representation with analogue properties rather than rely on a mere linguistic propositional representation [17]–[20]. By including blind individuals we can dissociate between supramodal and multimodal representations of spatial language. In case of a supramodal representation both blind and sighted individuals would show the same activation independently from the sensory modality that conveys spatial information. Namely, spatial information, available in visual and nonvisual modalities, is represented modality-independent. In case of a multimodal representation we would expect to find differences in neural activation for blind and sighted individuals, since both groups would recruit the network most suitable and available for the task. This would mean that the blind group would recruit a tactile/auditory network while the sighted would recruit a visual network. In addition to distinguishing between supramodal and multimodal representations the results from the blind participants also provide information about the functional development of this neural activation. If the left SMG is indeed activated by a supramodal representation of spatial language, i.e. this area is also activated in the blind, this demonstrates that the role of the left SMG is hard-wired and does not require visual experience.

Research with blind and sighted individuals has shown that there are only subtle differences in performance on several spatial tasks [17], [21], [22], which suggests that there might be overlap in the neural networks employed and that these functions also develop in the absence of vision. Indeed, such overlap in neural networks has been found in dorsal and ventral occipito-parietal areas [8], [10], [23]–[25]. Yet, the literature reports both similar and different findings on the connectivity within these networks in blind and sighted individuals, as well as on the strategies used. In a visual and/or tactile spatial one-back recognition task of 2D and 3D matrices sighted and blind individuals similarly activated a fronto-parieto network comprising bilateral posterior parietal cortex and dorsolateral and inferior prefrontal areas. These results indicate that visual experience is not a prerequisite for the development of spatial working memory [8], [10]. Vanlierde et al. [24] also found a similar pattern of activation for blind and sighted participants in a spatial imagery task of 2D matrices, involving the precuneus, superior parietal lobule and occipital gyrus, however, participants differed in their strategy. Sighted participants used a visual imagery strategy, while blind participants used an X-Y coordinate strategy [24], [26]. Whereas Stilla et al. [23] also observed a similar network for blind and sighted individuals in a tactile microspatial discrimination task, the effective connectivity differed between the blind and sighted. The findings by Stilla et al. can easily be explained by a supramodal representation of spatial information. Information derived from different modalities or different strategies, contains spatial properties that evoke a supramodal, modality-independent, representation yielding similar results in blind and sighted [11], [27]. At the same time, there may also exist subtle differences, because participants partly maintain the original traces of the input modalities, with sighted still having access to prior visual information, while the blind only have access to nonvisual information.

When comparing blind and sighted individuals it is important to keep in mind that the primary visual cortex of early blind people, who have been blind since they were at most four years old, has received very little or no visual input, and is therefore subject to neuroplastic changes resulting in reorganization [28]–[31]. As a consequence, the primary visual cortex of early blind people may get involved in performing non-visual tasks for which sighted people do not show any primary visual cortex activity. For example a wide variety of linguistic tasks have shown occipital cortex activity in blind people, e.g. letting blind participants read Braille [32]–[38]. Moreover, when Cohen et al. [39] used transcranial magnetic stimulation (TMS) on the visual cortex, comprising BA 17, 18, 19, the performance of blind participants decreased on Braille symbol identification. In contrast, sighted participants did not decrease in their performance on embossed Roman letter identification during TMS stimulation to the visual cortex, also comprising BA 17, 18, 19 (see [30] for a review). The primary visual cortex of early blind people is not only involved in tactile reading tasks, but also in verbal word association tasks indicating that the functionality of the reorganization goes beyond the analysis of tactile information [40]. Covertly generating an associated verb to a noun has been shown to elicit primary visual cortex activation in blind participants but not in sighted participants, supporting the functional role of this activation accuracy was reduced during repetitive TMS [32], [41]. In addition, the reorganized primary visual cortex does not only show activity related to language processing, but also activity related to spatial imagery [24] and tactile discrimination [42], [43] tasks. Learning to discriminate the orientation of a letter *T* applied to the tongue with a tongue display unit (TDU) resulted in significant visual cortex activation in blind, but not in sighted individuals. In a follow-up study TMS was used to stimulate the visual cortex before and after training with the TDU [44]. Kupers et al. [44] found that blind participants reported tactile sensations on the tongue. Interestingly, these sensations were somatotopically organized.

The foregoing results suggest the possibility that the reorganized visual cortex in the blind is also specifically suitable for processing spatial information. In the context of our spatial language experiment we were interested in the functional relevance of the expected visual cortex activation in the blind. Hamilton et al. [45] reported on a congenitally blind patient who suffered bilateral occipital strokes. She was a profound Braille reader, but after the stroke she was unable to discriminate tactile information necessary for the complex spatial decoding involved in Braille reading. The involvement of the visual cortex in spatial discrimination was supported by a low-frequency rTMS study by Merabet et al. [46]. They found that rTMS to the visual cortex specifically impaired distance, but not roughness, judgments. The hypothesized additional occipital activation in blind during the spatial language task could follow from language processing in general, comparable to what has been found with other language tasks (such as the classic verb generation task), or demonstrate specific involvement in processing language pertaining to space. Nevertheless, the findings on the spatial language task only allow backward inference about any expected activation in the occipital cortex, i.e. comparing them to previous findings from other tasks. To test to what extent the reorganization is functional and comparable to the established body of literature on reorganization, the same participants also performed a covert verb generation task.

In light of the foregoing, the aim of the present study was twofold: first, to determine whether left SMG activation is associated with supramodal representations which develop in the absence of visual experience; second, to investigate the possible functional reorganization of spatial language processing in the blind. The current task was adapted from Noordzij et al. [4] to an auditory presentation and included different sentence types in order to determine the specificity of the left SMG.

## Materials and Methods

### Participants and Ethics

Fourteen early blind and fourteen blindfolded sighted control participants, with no neurological or motor deficits, participated in this experiment. One early blind and one sighted control participant were excluded from the analysis due to task performance at chance level, resulting in two groups of thirteen participants. There were twelve congenitally blind participants, who have been blind since birth. One participant was born with buphtalmus and lost his eyes in an operation at the age of four. However, before that age he was severely visually impaired and he has no memory of vision. The blind participants and healthy controls were matched for sex, education, age and handedness (for details and etiology of the blind participants see Table 1). All participants signed an informed consent prior to the experiment, which was approved by the Medical Ethical Board (Medische-etische toetscomissie (METC-protocolnumber 05/186-E)).

**Table 1:** Description of Early Blind participants.

*Subject number*	*Occupation*	*Education level*	*Sex*	*Handedness*	*Age*	*Etiology* *	*Age of onset*
1	Unemployed	University	f	r	39	ROP	0
2	Translator	Higher Education	f	a	35	ROP	0
3	Computer Programmer	Higher Education	m	r	38	CG	0
4	Sports Masseuse	Vocational Education	f	a	45	LCA	0
5	Office Assistant	Higher Education	f	r	32	CG	0
6	Educator	University	m	l	40	A	0
7	Unemployed	Higher Education	f	l	31	LCA	0
8	Policy Worker	University	f	l	30	LCA	0
9	Student	Higher Education	m	l	19	LCA	0
10	Sales Person	Higher Education	m	a	41	ROP	0
11	Student	Higher Education	m	r	22	ND	0
12	Programmer	Higher Education	m	l	49	B	4
13	Sound Technician	Higher Education	m	r	53	ROP	0

*definitions of etiology: A  =  Anophthalmia, B =  Buphtalmus, CG  =  Congenital Glaucoma, LCA  =  Leber's Congenital Amaurosis, ND = Norrie Disease, ROP  =  Retinopathy of Prematurity. Mean age Early Blind: 36.46±9.80. Mean age Sighted Controls: 37.15±11.16.

### Design & Procedure

Both tasks were presented auditorily through MR-compatible headphones. The task was administered on a PC with Presentation software 9.90 (Neurobehavioral Systems, Albany, CA). Prior to the fMRI experiment participants were instructed about the scanning procedure and both tasks were explained. During the instruction phase participants practiced the two tasks and received feedback.

**Figure 1:**
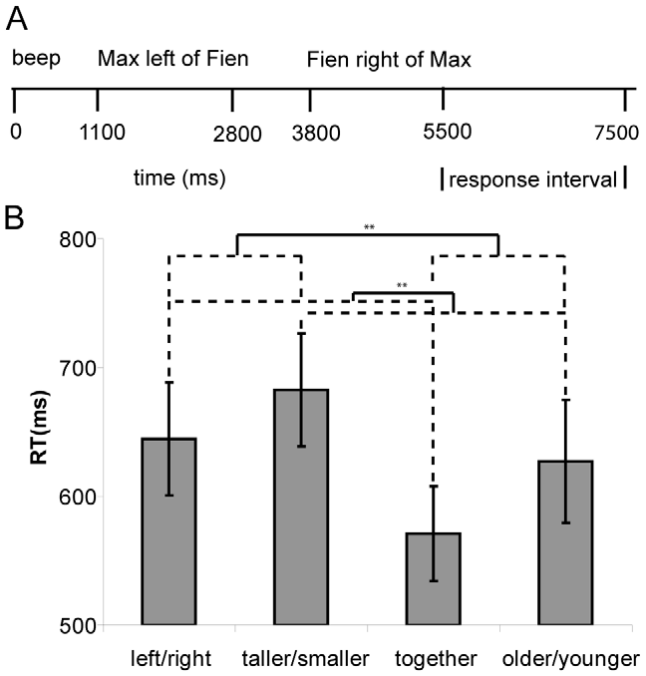
Example of a trial and behavioral results (RTs) from the spatial language task. (A) An example of a single trial in the spatial language task. Each trial starts with a beep and consists of two spoken sentences and a response interval. In this situation the response was affirmative. (B) Mean reaction time and standard errors in ms on the different sentence types, collapsed for blind and sighted participants. Significant (*p*<.05) effects for Space and Category are marked with **.

The spatial language task was based on a sentence verification paradigm. The trials consisted of a sequence of two auditorily presented sentences (see Figure 1A). The experiment included four sentence types with a spatial and non-spatial compound preposition and compound adverb (*left/right of, together with, taller/smaller than* and *older/younger than*). It is a matter of debate to what extent conjunctions, such as *together with,* contain a spatial element. Importantly, this type of sentence does not require imposing a reference frame and setting a spatial template in order to allow for an explicit comparison along a spatial dimension [47]. This is precisely the type of contrast we were interested in.

The task was to judge whether or not the two sentences described the same situation. All participants were instructed to finish listening to the entire second sentence before responding as quickly and as accurately as possible. Participants responded with the thumb of their right hand on a MR-compatible pneumatic response-box. They pressed the right button when they thought the trial was correct, and the left button when the trial was considered incorrect (see Table 2). There were three types of trials: repetitions, reversals and catch trials (see Table 2). A repetition trial consisted of two sentences in which the order of the two names was identical. In a reversal trial the order of the two names was different for the two sentences. Catch trials contained a third name (i.e. not mentioned in the first sentence) in the second sentence. The participants did not have to pay attention to the different trial types, but only to the different sentence types, which were separated in time by a short silent interval. There were 144 trials presented in blocks (15s) of two trials of one sentence type. Each sentence type consisted of 36 trials, with 16 reversals, 16 repetitions and 4 catch trials. There were four sessions of 18 blocks. The inter-block interval varied between 6s and 9s. The different sentence types were presented pseudo-randomly. No feedback was given.

**Table 2:** Five different options for sentence 2 in relation to sentence 1.

Sentence Option(e.g. S1 is ‘Fien taller than Max’)	Trial Type	Correct Response
Relation and position of names identical	Repetition	yes
*S2: ‘Fien taller than Max’*		
Relation changed and position of names identical	Repetition	no
*S2: ‘Fien smaller than Max’*		
Relation and position of names changed	Reversal	yes
*S2: ‘Max smaller than Fien’*		
Relation identical and position of names changed	Reversal	no
*S2: ‘Max taller than Fien’*		
Introduction of a third name	Catch	no
*S2: ‘Fien taller than Stein’*		

The verb generation task consisted of auditorily presented nouns and participants had to covertly generate an associated verb [32], [40], [41], [48]. Since giving a verbal response during scanning produces movement artifacts the participants were required to only give a mental response. Therefore, no behavioral data were collected.

The verb generation task consisted of three conditions: *word*, *nonword* and *rest*. In the *word* condition participants heard a noun and were instructed to covertly generate an associated verb. The *nonword* condition consisted of trials with passive listening to reversed speech. The sound-spectrum of regular words was reversed with the program CoolEdit 2000 (www.cooledit.com). In this condition the stimuli sounded like words, but had no semantic interpretation, which was used as a control condition for auditory input [49].

We used a block-design with 18 blocks of 10 trials of 2.8s, 6 blocks of each condition (*word, nonword* and *rest*), presented in pseudorandom order. At the beginning of each block with sound stimuli a beep was presented. A short beep indicated a block with words while a long beep indicated a block with non-words. No feedback was given.

### MR Data Acquisition

Scanning was performed with a 3.0T Philips Achieva scanner using an eight-channel SENSE headcoil to acquire T2^*^-weighted images with blood oxygenation level dependent (BOLD) contrast. We used the principles of echo shifting with a train of observations (PRESTO) scanning sequence, combined with 2D-SENSE acquisition. This sequence uses three dimensional imaging in combination with a delayed echo read-out after the next RF pulse [50]. Also, T2* acquisition was accelerated in 2 directions (2D-SENSE) by skipping lines in K-space. Together this resulted in a four-fold increase in imaging speed in PRESTO. PRESTO-SENSE has been demonstrated to yield fast and reliable activation for 1D-SENSE [51], and is even more sensitive than conventional EPI when using 2D-SENSE [50]. An entire volume was acquired in 500.3 ms (TE = 32.4 ms, TR = 21.75 ms, flip angle = 10°, 56×64 acquisition matrix, 32 sagittal slices, isotropic voxels of 4 mm, FOV(ap,fh,rl)  = 224×256×128 mm and a SENSE factor of 2 in the AP and 1.8 in the LR direction). Each scanning session was preceded by ten dummy volumes in order to accomplish steady state transversal magnetization.

The spatial language task consisted of four sessions of 800 volumes each. After the final session a reference-scan was acquired, with a flip angle of 25°, but otherwise identical to the PRESTO-SENSE functional MRI images. Due to the increased flip angle this image had slightly more anatomical contrast and was used for coregistration with the anatomical scan. After the spatial language task a T1-weighted anatomical scan was acquired (TE = 4.6 ms, TR = 9.86, flip angle = 8°, 224×224 acquisition matrix, 160 coronal slices, voxel size = 0.875×0.875×1 mm and FOV(ap,fh,rl)  = 224×160×168 mm). During the anatomical scan the participants could rest.

The same PRESTO-SENSE sequence was used to acquire T2^*^-weighted images with blood oxygenation level dependent (BOLD) contrast. The verb generation task consisted of one session of 1024 volumes. After the task another reference scan with slightly more anatomical contrast was collected.

### Data Analysis

#### Behavioral Data

For each participant individual mean reaction times and performance scores in the spatial language task were collected. Group analyses were performed with SPSS (SPSS for Windows, Rel. 14.0.2. 2006. Chicago: SPSS Inc.). Behavioral data were analyzed with a 2×2×2×2 mixed Analysis of Variance (ANOVA). Space (spatial/non-spatial), Category (compound preposition/compound adverb) and Trial Type (repetition or reversal) were the within-subject factors and Group (blind or sighted) was the between-subject factor. The catch trials were excluded from the analysis: they were included (11%) to keep participants alert and to make sure they paid attention to both names mentioned in the sentence. The results reported below show the effects for correct answers with a significance level of *p*≤0.05. When pairwise comparisons were tested the significance level was corrected for multiple comparisons using the Bonferroni method. SPSS multiplies the *p*-value with the Bonferroni multiplier instead of dividing α by the Bonferroni multiplier. However, the results are equal and we will denote the Bonferroni corrected *p*-values by *p_B_*_._

Since the verb generation task required participants to covertly generate associated verbs there were no behavioral data which were analyzed.

#### Functional Imaging Data

Imaging data were analyzed with SPM5 (Wellcome Department of Imaging Neuroscience, Institute of Neurology, London, http://www.fil.ion.ucl.ac.uk/spm/software/spm5/) and the MarsBaR toolbox for SPM5 (http://marsbar.sourceforge.net/) running under Matlab (R2007b, The Math Works, Inc., Natick, MA). Preprocessing included coregistration and realignment. The anatomical scan was segmented and spatially normalized with medium regularization (0.01). The spatial normalization parameters from the ‘unified segmentation’ routine were used to normalize all functional scans [52], [53], which were then spatially smoothed with a kernel of 8 mm FWHM.

First level statistics was performed for each participant individually. A high-pass filter with a cutoff period of 128s was applied to remove low frequency fluctuations. The model for the spatial sentence comprehension task contained 62 regressors. The design matrix consisted of eight regressors (Space × Category × repetition and reversal trials), three or four nuisance-regressors for the sporadic catch-trials in each session and three additional nuisance-regressors for each session to filter out a very systematic scanner-related oscillation in a very narrow frequency band exactly at 0.5 Hz. Each functional regressor was convolved with a hemodynamic response function.

Using a general linear model the parameter estimates were calculated for all brain voxels. Several effects, mentioned below, were tested by means of linear contrasts between the parameter estimates for different conditions. These contrast images were then passed to a second-level analysis, to model any group effects.

The contrast of interest in the spatial language task were: (i) general language and reorganization effects: *task activation > rest* and (ii) specific language effects: (a) *left/right* > *together*, (b) *spatial (left/right and taller/smaller) > non-spatial (together and older/younger)*. Spatial prepositions can be divided into directional prepositions indicating a change in direction, and locative or relational prepositions, describing relations between objects [54]. Here we extend the relational aspect of spatial prepositions. The distinction between spatial and non-spatial might not be as straightforward as assumed. The three sentence types *left/right of*, *taller/smaller than* and *older/younger than* can all be represented on an imaginary axis. *Left/right* can be ordered on a horizontal axis, *taller/smaller* on a vertical axis and *older/younger* on a horizontal time line. The control condition *together with* does not automatically generate such a line analogy. Therefore, we included a third contrast (c) *relational > together* to test this analogy (for the (a), (b) and (c) contrasts the results for repetition and reversal trials were collapsed). In a fourth contrast we tested the difference between (d) *reversal > repetition*.

In order to determine the differences between the blind and sighted we performed two-sample t-tests at the second level with a contrast between blind and sighted individuals. The commonalities between the two groups were tested by means of a conjunction analysis that tested the conjunction null hypothesis over two orthogonal contrasts [55], [56]. *P*-values were adjusted for the search volume using random field theory and inferences were drawn at the cluster level (details are explained below). The null distribution for the minimum statistic was based on two statistics. This enabled us to infer a conjunction of activation in an area in both blind and sighted groups [55].

We report the results of a random effects analysis, with inferences drawn at the cluster level. *P*-values were corrected for multiple comparisons by combining a *p*<0.001 voxel-level threshold with a cluster extend threshold to obtain a *p*<0.05 whole-brain corrected significance level [57]. For the specific language effects a small volume correction was applied [58]. This procedure constrained our search space to a spherical region of interest (ROI) in the left SMG (a radius of 10mm around −36, −48, 40 MNI coordinates, based on the coordinates from the peak voxel of a significant cluster of 18 voxels reported by Noordzij et al. [4]).

The model of the verb generation task consisted of two regressors for the *word* and *nonword* condition and three additional nuisance-regressors to filter out a very systematic scanner-related oscillation in a very narrow frequency band exactly at 0.5 Hz. The two functional regressors were convolved with a hemodynamic response function.

The contrast of interest in the verb generation task was the analysis: *word* > *nonword*. In order to determine the differences between the blind and the sighted we performed a two-sample t-test at the second level with a contrast between blind and sighted individuals. The commonalities between the two groups were tested by means of a conjunction analysis. We report the results of a random effects analysis, with inferences drawn at the cluster level, with similar correction applied as in the spatial language task.

## Results

### Spatial Language Task

#### Behavioral Data

The behavioral data indicated that participants performed very accurately on the spatial language task (>90% correct trials). A 2(Space) ×2(Category) ×2(Trial Types) ×2(Group) mixed ANOVA on the percentage of correct trials, revealed no group differences between the blind and sighted participants (*F*(1,24) <1). There was a significant main effect of Space. Participants performed slightly better on the non-spatial sentences (97%±1.0 correct), compared to the spatial sentences (96%±1.1 correct, *F*(1,24)  = 4.38, *p* = .047). There was also a significant main effect of Trial Type. Participants made slightly, but significantly, more errors on the reversal sentences (95%±1.5 correct), compared to the repetition sentences (98%±.7 correct, *F*(1,24)  = 7.19, *p* = .01).

The behavioral results on the mean response times are shown in Figure 1B. There was a significant main effect of Space (*F*(1,24)  = 18.24, *p*<.001), Category (*F*(1,24)  = 6.73, *p*<.016), and Trial Type (*F*(1,24)  = 15.95, *p* = .001). The interaction between Category and Trial Type was also significant (*F*(1,24)  = 5.61, *p* = .026). Pairwise comparisons between repetitions and reversals for both categories showed that participants evaluated repetitions significantly faster than reversals in all conditions (prepositions: *t*(11)  = −2.88, *p_B_* = .008, adverbs: *t*(11)  = −3.51, *p_B_* = .002), but the difference was larger for adverbs than prepositions.

Although there was no main effect of Group (*F*(1,24) <1), the interaction between Group and Trial Type was significant (*F*(1,24)  = 5.85, *p* = .026). Further analysis indicated that blind participants were significantly slower on reversal than repetition sentences (*t*(11)  = −4.53, *p_B_*<.001), while the sighted participants were equally fast on both sentence types (*t*(11)  = −1.11, *p_B_* = .276).

### Spatial Language Task

#### Functional Imaging Data

**Figure 2:**
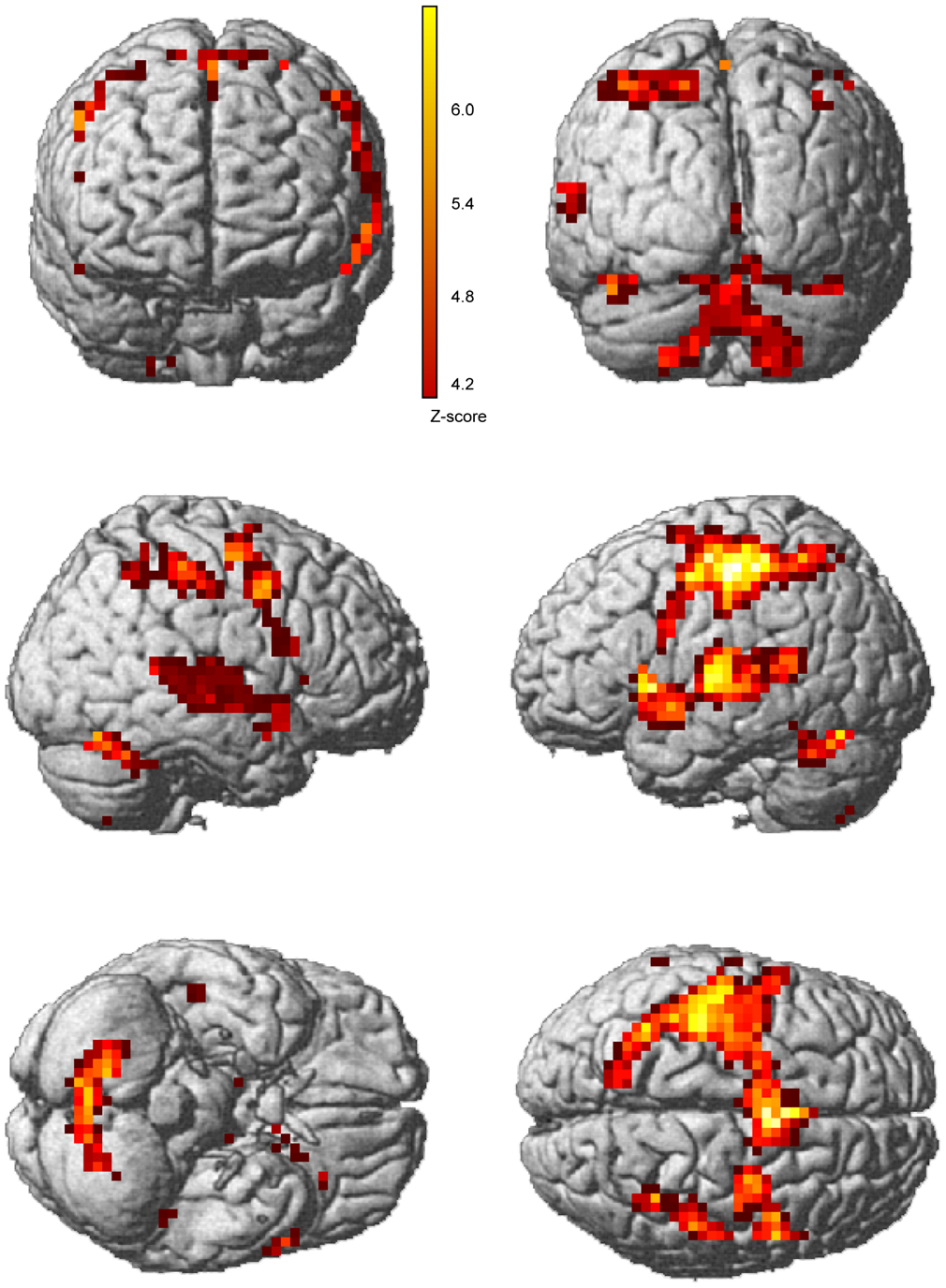
fMRI results from the spatial language task, commonalities between blind and sighted. Network of general task related activation in blind and sighted during the spatial language task. A whole brain conjunction analysis of the contrast *task activation > rest.* T-threshold = 4.2, spatial extent threshold: >10 voxels.

The neuroimaging results focus on four main contrasts in the spatial language task. The general task activation was analyzed by means of a whole brain conjunction analysis on the contrast *task activation > rest*. The network of significant activation in blind and sighted comprised bilateral parietal areas, bilateral thalamus, right cerebellum and right lingual gyrus (see Table 3 for details and Figure 2 for a visual representation).

**Table 3:** Spatial language general task activation compared to rest common to blind and sighted subjects.

Region	Cluster	Peak voxel	MNI coordinates (mm)		
	Size	Z-score	x	y	z
R cerebellum	472	5.97	32	−52	−28
		5.51	20	−55	−20
		5.19	12	−60	−16
L postcentral gyrus	655	5.96	−36	−28	52
L inferior parietal lobule		5.82	−36	−52	52
L supplementary motor area		5.71	0	4	56
R superior temporal gyrus	136	5.64	52	−24	0
		5.15	52	−36	4
		5.05	52	−12	4
L superior temporal gyrus	373	5.42	−56	−16	0
L insula		5.40	−36	16	4
L superior temporal gyrus		5.10	−52	4	−12
R thalamus	192	5.37	12	−16	8
		5.04	12	−28	−4
L thalamus		4.98	−12	−16	0
R precentral gyrus	112	5.16	48	4	44
		5.03	36	−12	60
		4.89	28	−4	52
R postcentral gyrus	102	5.01	44	−28	44
		4.95	36	−32	44
R inferior parietal lobule		4.47	36	−55	48
R inferior frontal gyrus	16	5.16	32	20	4
R lingual gyrus	15	4.71	4	−76	4
		4.51	12	−72	12

L  =  left, R  =  right. T-threshold = 4.20, spatial extent threshold: >10 voxels.

**Figure 3:**
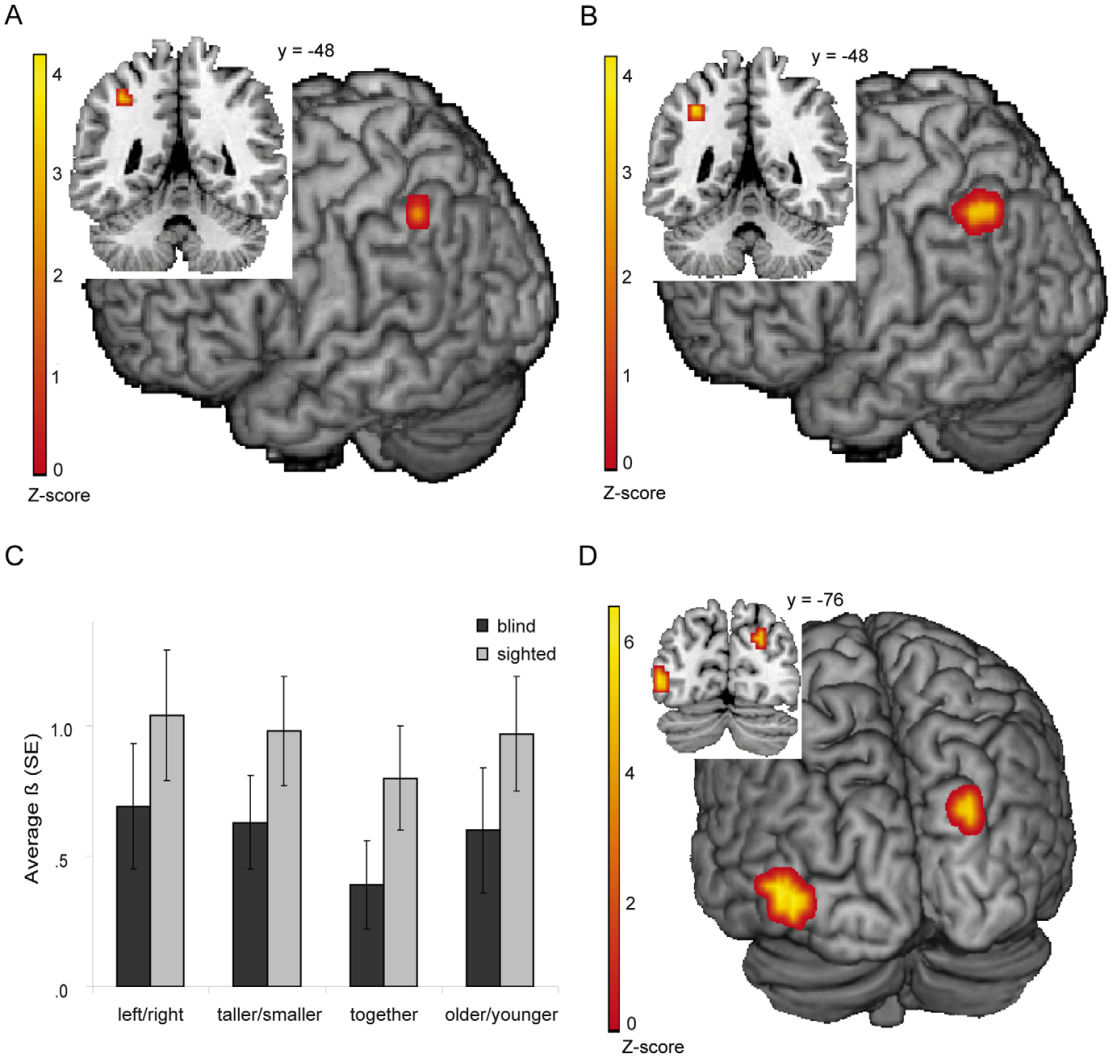
fMRI results from the spatial language task, commonalities and differences between blind and sighted. (A) ROI conjunction analysis of the contrast *left/right* > *together* showing a significant cluster of activation in the blind and sighted individuals. T-threshold  = 2.6, spatial extent threshold: >5 voxels, peak voxel MNI coordinates: −36, −48, 44, Z-score  = 2.80, cluster size: 6 voxels. (B) ROI conjunction analysis of the contrast *relational (left/right, taller/smaller and older/younger)* > *together* showing a significant cluster of activation in the blind and sighted individuals. T-threshold  = 2.6, spatial extent threshold: >5 voxels, peak voxel MNI coordinates: −36, −48, 44, Z-score  = 3.20, cluster size: 15 voxels. (C) The average parameter estimates (ß) for the functional ROI of the left SMG from the contrast *relational* > *together*. The dark gray bars indicate the parameter estimates for the blind and the light gray for the sighted individuals. The error bars denote the standard error of mean. (D) Difference between blind and sighted individuals for the contrast *task activation > rest* in the spatial language task. T-threshold  = 4.2, spatial extent threshold: >10 voxels. A cluster of 23 voxels: middle occipital gyrus (peak voxel MNI coordinates: −48, −80, 4, Z-score  = 4.66). A cluster of 10 voxels: cuneus (peak voxel MNI coordinates: 24, −80, 32, Z-score  = 4.17).

Further analyses were based on a previous fMRI study in sighted individuals [4]. For the contrast spatial preposition *left/right* versus non-spatial preposition *together* an ROI conjunction analysis was performed which resulted in a significant cluster of activation in both blind and sighted individuals in the left SMG (T-threshold  = 2.6, spatial extent threshold: >5 voxels, peak voxel MNI coordinates: −36, −48, 44, Z-score  = 2.80, cluster size: 6 voxels, see Figure 3A). We thus replicated the findings by Noordzij et al. [4] and extended them to blind participants. The present study aimed to investigate the specificity of this finding by adding a spatial and non-spatial adverb to the stimulus set. The ROI conjunction analysis on the *spatial > non-spatial* contrast did not yield any significant results.

However, as stated before, the sentence type *older/younger* can be considered spatial when represented on a horizontal axis. One can imagine comparing ages of people on a timeline, which, as space, can be represented by a canonical axis. We therefore analyzed the contrast between relational sentence types (*left/right*, *taller/smaller* and *older/younger*) and the non-relational sentence type *together*. The conjunction analysis on this *relational > together* contrast also revealed a significant cluster in both blind and sighted individuals in the left SMG (T-threshold  = 2.6, spatial extent threshold: >5 voxels, peak voxel MNI coordinates: −36, −48, 44, Z-score  = 3.20, cluster size: 15 voxels, see Figure 3B).

The regression parameters of the left SMG for all four sentence types (Figure 3C), in both the blind and sighted participants, were higher for sentences in which an evaluation about a relation (*left/right, taller/smaller* or *older/younger*) was required than for sentences in which only the correspondence of the two names had to be verified (a sentence with *together*).

The behavioral results showed that the responses of blind participants were significantly slower for reversals than repetitions, while the sighted participants responded equally fast to both. However, there was no significant difference in activation in the left SMG between the reversals and repetitions for both blind and sighted individuals, nor for the blind in particular.

Apart from commonalities the differences between blind and sighted were also analyzed to determine the level of reorganization. For the whole-brain analysis on the general contrast *task activation > rest* we found a significantly higher activation for the blind compared to the sighted individuals in the left middle occipital gyrus and right cuneus (see Figure 3D for details). There were no significantly greater activations in the sighted, compared to the blind. Furthermore, there were no general task activation differences between blind and sighted, tested in both directions, in the left SMG.

### Verb Generation Task

#### Functional Imaging Data

The verb generation task was included in order to determine the level of reorganization for general language processing. A conjunction analysis between the blind and sighted participants on the contrast *word > nonword* revealed those areas that are specific to generating verbs. In blind and sighted participants covert language generation activated bilateral language areas, including the inferior frontal gyrus and middle temporal gyrus also known as Broca's and Wernicke's area respectively (see Table 4 for details). Further significant activation was found bilaterally in the cerebellum and the supplementary motor area as well as the left precentral gyrus. These latter areas are involved in the covert generation of the words [59], [60].

**Table 4:** Verb generation general language processing effects common to blind and sighted subjects.

Region	Cluster	Peak voxel	MNI coordinates (mm)		
	Size	Z-score	x	y	z
Supplementary motor area	127	5.40	0	0	60
		4.69	0	16	44
		4.53	12	0	68
R Cerebellum	34	4.84	32	−64	−24
		4.67	40	−60	−28
L inferior frontal gyrus	123	4.83	−48	16	−4
		4.68	−20	12	4
		4.52	−52	16	4
L precentral gyrus	21	4.82	−48	−4	48
R inferior frontal gyrus	48	4.65	36	24	4
		4.51	48	16	−4
L middle temporal gyrus	33	4.46	−52	−36	0
		4.42	−56	−24	0
L superior temporal gyrus		4.08	−60	−52	8
L cerebellum	36	4.31	−40	−56	−24
		4.24	−36	−48	−24
		4.08	−32	−60	−24
R cerebellum	22	4.28	12	−68	−16
		3.92	8	−60	−8

L  =  left, R  =  right. T-threshold  = 4.20, spatial extent threshold: >10 voxels.

**Figure 4:**
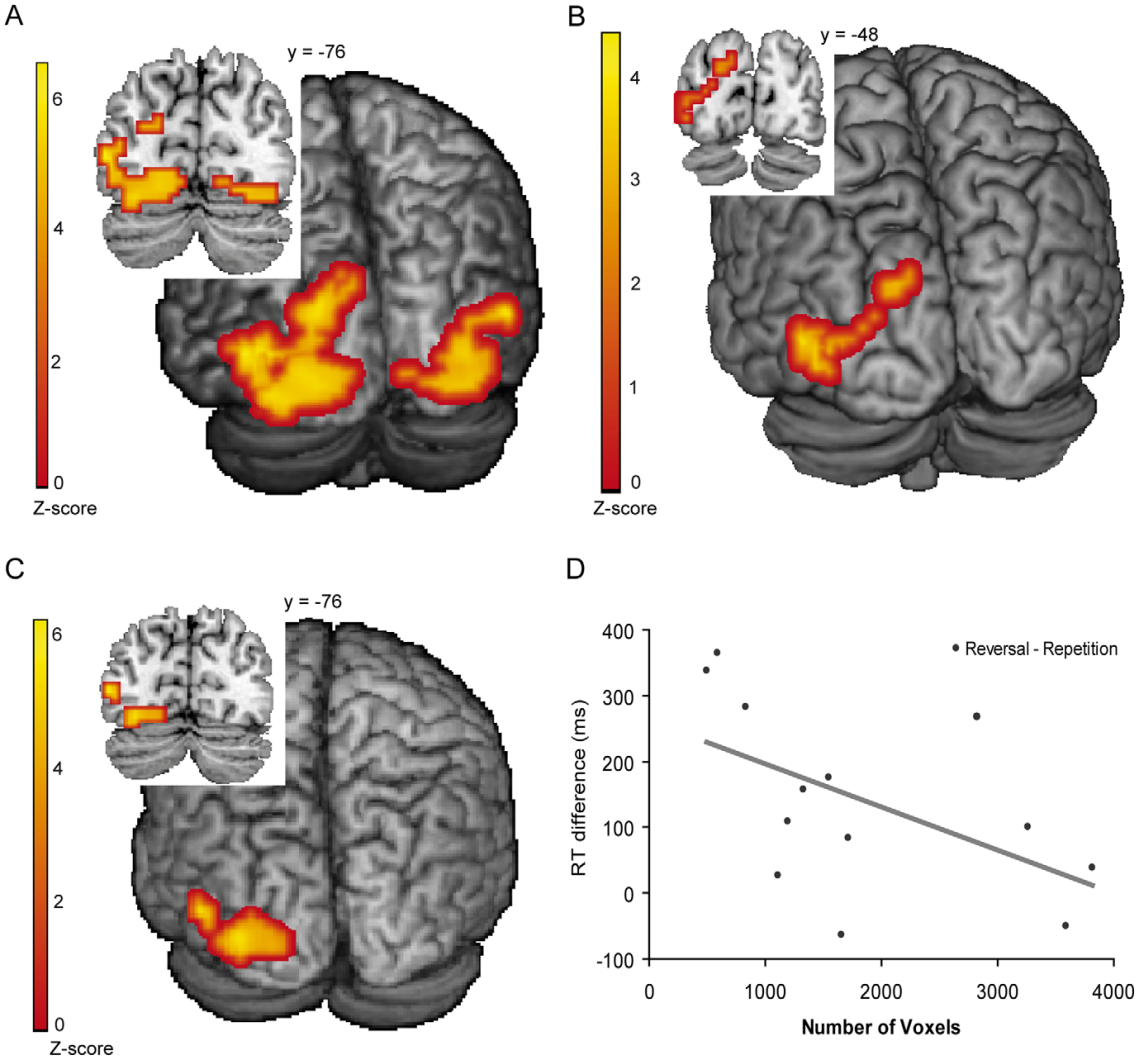
fMRI results from the verb generation task and reorganization analysis. (A) Difference between blind and sighted individuals for the contrast *word > nonword* in the verb generation task. T-threshold  = 4.2, spatial extent threshold: >10 voxels. Left cluster of 195 voxels: middle occipital gyrus (peak voxel MNI coordinates: −48, −76, 4, Z-score  = 4.86); cuneus (peak voxel MNI coordinates: −48, −76, 4, Z-score  = 4.86). Right cluster of 94 voxels: middle temporal gyrus (peak voxel MNI coordinates: 56, −56, 4, Z-score  = 4.83); middle occipital gyrus (peak voxel MNI coordinates: 40, −60, −8, Z-score  = 4.34). (B) Difference between blind and sighted individuals for the contrast *task activation > rest* in the spatial language task within the verb generation ROI. T-threshold  = 3.5, spatial extent threshold: >5 voxels. A cluster of 31 voxels: middle occipital gyrus (peak voxel MNI coordinates: −48, −76, 4, Z-score  = 4.52). A cluster of 7 voxels: cuneus (peak voxel MNI coordinates: −16, −84, 28, Z-score  = 3.92). (C) Difference between blind and sighted individuals for the contrast *reversal > repetition* in the spatial language task within the verb generation ROI. T-threshold  = 3.5, spatial extent threshold: >5 voxels. A cluster of 14 voxels: the left middle occipital gyrus (peak voxel MNI coordinates: −48, −76, 4, Z-score  = 4.55, cluster size: 14 voxels) A cluster of 51 voxels: left lingual gyrus (peak voxel MNI coordinates: −32, −72, −12, Z-score  = 4.38, cluster size: 51 voxels. (D) Correlation between the number of activated voxels in the reorganized occipital lobe in the blind and the difference in RT (ms) between reversals and repetitions (r(11)  = −.54, p = .03).

The differences between blind and sighted participants were found mainly in the occipital cortex (see Figure 4A for details). The left cuneus and bilateral middle occipital gyrus showed significantly higher activation for blind compared to sighted participants. In the right hemisphere the activation of the middle occipital gyrus extended slightly into the middle temporal gyrus.

### Reorganization

The *word > nonword* contrast in the verb generation task revealed reorganized areas in the blind, similar to what has been previously found [41], [48]. The functional result from the verb generation task was used to define an ROI for further analysis on the reorganization within the spatial language task. For the general contrast *task activation > rest* we found a significantly higher activation for the blind compared to the sighted individuals in the left middle occipital gyrus (T-threshold  = 3.5, spatial extent threshold: >5 voxels, peak voxel MNI coordinates: −48, −76, 4, Z-score  = 4.52, cluster size: 31 voxels) and left cuneus (T-threshold  = 3.5, spatial extent threshold: >5 voxels, peak voxel MNI coordinates: −16, −84, 28, Z-score  = 3.92, cluster size: 7 voxels, see Figure 4B for details). The reorganized areas did not show a modulation for the *left/right > together* contrast, neither for the *spatial > non-spatial* contrast, nor for the *relational > together* contrast. Interestingly, the contrast *reversal > repetition* did show significant activation within the reorganized areas in the blind. The activation was found in the left middle occipital gyrus (T-threshold  = 3.5, spatial extent threshold: >5 voxels, peak voxel MNI coordinates: −48, −76, 4, Z-score  = 4.55, cluster size: 14 voxels) and left lingual gyrus (peak voxel MNI coordinates: −32, −72, −12, Z-score  = 4.38, cluster size: 51 voxels, see Figure 4C). This finding suggests that the reorganized areas are involved in processing information with a higher linguistic load, which has been reported before.

For example, Amedi et al. [48] showed that superior verbal memory is correlated with occipital cortex activation, while Van der Lubbe et al. [61] have shown that superior duration discrimination abilities were related to enhanced occipital negativity in the blind, during an electroencephalogram (EEG) experiment. We performed a correlation analysis on the spatial language data in order to test this explanation. For each blind participant the number of significantly activated voxels, within the verb generation ROI, on the contrast *task activation > rest* was counted. There was a significant negative correlation between the number of activated voxels in the reorganized area and the difference in reaction time between reversals and repetitions (*r*(11)  = −.54, *p*  = .03, see Figure 4D). This means that blind participants who were better at the reversal trials, i.e. responded equally fast to reversals and repetitions, were most likely to have a higher level of reorganization.

## Discussion

The aim of this study was to determine whether spatial language is represented in a supramodal representation in the left SMG that does not require visual experience to develop its functionality. Sustained activation in the left SMG in blind and sighted participants during an auditory spatial language task supported a supramodal representation of spatial language. In addition, the verb generation task further established the reorganized cortical areas in the blind. Activation in reorganized visual cortical areas in the blind was not associated with specific spatial processing. However, this activation did have functional relevance because it increased with higher linguistic load.

Besides differences in the occipital lobe most of the activated areas were similar in the blind and sighted individuals. The large language network that was revealed in the verb generation task indicated that reorganization in the blind was limited to the occipital areas. This finding was supported by the large network found in the spatial language task for both blind and sighted as well. The overlapping result in the left SMG during the spatial language task also supports this notion. The present study used an auditory presentation and extends the finding by Noordzij et al. [4] on the contrast *left/right* > *together*.

Noordzij et al. [4] used a visual paradigm with verbal and visual-spatial contexts. In their study the left SMG was activated regardless of the nature of the visual stimulus. The first stimulus was always a sentence while the second stimulus could be another sentence or a picture. Noordzij et al. analyzed activation related to the second stimulus separately and found sustained left SMG activation for both types of second stimuli. In contrast, the behavioral results revealed that participants responded significantly faster to pictures compared to sentences indicating that sighted participants used a spatial representation that was most likely visual, to compare a spatial sentence to a picture [4], [62]. If the activation found in the left SMG had been due to linguistic processing of spatial sentences, then no sustained activation for the second stimulus, in particular the picture, should have been found. Instead the maintained activation found in the left SMG for both verbal and visual-spatial context might support a supramodal representation of spatial information.

Interestingly, the auditory paradigm in the current study generated left SMG activation similar to the visual paradigm used by Noordzij and colleagues. Moreover, while there were subtle behavioral differences between the blind and sighted participants, the blind also activated the left SMG and the behavioral differences were not correlated to left SMG activation. The results from the conjunction analysis are based on the conjunction null hypothesis stating that the effect was present in both groups. This analysis does not tell us whether the activation in both groups is actually similar. However, the presence of left SMG activation in the blind is a strong indication that it is not related to visual processing. In order to verify that the activation in the left SMG is indeed supramodal a visual and auditory paradigm should be combined in a single experiment with sighted individuals. The results from the current experiment imply that the activation in the left SMG does not depend on visual experience, nor on the nature of the stimulus or the sensory input channel being used, but rather suggests a superordinate level of processing with maintained traces to the input modality and thus is associated with the supramodal representation of spatial terms.

Importantly, the activation of the left SMG in the blind participants, who have never received visual input, demonstrates that visual experience is not a prerequisite for developing a supramodal representation of spatial information. This finding is in line with recent findings on object and spatial processing in cortical areas that were previously thought to be involved in encoding visual information only [8]–[10], [25], [63]. For example, the motion-sensitive middle temporal cortex (hMT+) not only responds to optic flow, but also to tactile flow, auditory movement or movement *per se* [42], [64], [65]. More recently, a supramodal sensory representation has also been found for the mirror system. This supramodal mirror system develops without access to visual experience and allows blind individuals to interact effectively with the world around them [66].

The findings discussed above suggest that vision is not crucial to build up these supramodal representations, but an interesting question that remains is whether automatic transfer to the visual modality is possible. Several case-studies on congenitally blind patients with restored vision reported that they had profound difficulties with visual perception [67], [68]. They were able to distinguish between objects, but could not identify them. In order to ‘tell’ what the object was, the new visual input had to be matched to the established supramodal representation. In a recent match-to-sample experiment performance was poor when subjects with restored vision had to visually match a haptically explored object [69]. Although there was no direct transfer of spatial information, mapping of the visual input to the supramodal representation developed rapidly. This suggests that vision is not a prerequisite for building up spatial representations, but a certain degree of calibration of the visual system is needed before the link between the visual input and the supramodal representation can be established.

In addition to replicating the *left/right > together* contrast from Noordzij et al. [4] the *relational* > *together* contrast also showed significant activation in the left SMG. Space and time are closely related, as described by the Metaphoric Mapping Theory [2], [70]–[73], which states that spatial schemas and temporal schemas share a relational, basic spatial structure. This allows organization of temporal concepts. Santiago et al. [73] have shown that there exists a space-time conceptual metaphor along a mental time line: left-past and right-future. Accordingly, the temporal sentence type *older/younger than*, used in the current study, could also have been analyzed within a quasi-spatial schema along a mental time line. As a result there were three relational sentence types which ordered information along a canonical axis. We suggest that the left SMG might be involved in the discrimination of information ordered along a canonical axis, instead of being selectively involved in processing spatial prepositions.

The current study tested brain activation in two very different tasks. One might wonder why it is relevant to include the verb generation task. As mentioned in the introduction we were interested in the functional reorganization of the occipital cortex especially during the spatial language task. A recent review by Kriegeskorte et al. [74] explained the possible problems of using the same data for selection and selective analysis, i.e. using the difference between blind and sighted on the *task activation > rest* contrast in the spatial language task to determine the ROI for testing the *relational > together* contrast could yield distortions and invalid statistics. The verb generation task, however, is inherently independent of the spatial language task. As such it was useful as a localizer for language related areas in the blind and provided an independent ROI which allowed for further analysis of occipital cortex activation in the spatial language task.

There is a large body of literature on the application of the covert verb generation task and it has proven to elicit robust language related activity as well as reveal occipital areas that have been subjected to reorganization in the blind [32], [40], [41], [48], [75]-[77]. The results from the verb generation task verified that the blind activated classical language areas, similar to the sighted participants. Importantly, the blind also showed additional language related activity in the occipital cortex for two very different tasks: the verb generation and the spatial sentence comprehension task. This constitutes further evidence of reorganization of the occipital cortex and adds to the established body of literature on reorganization. There is an increasing amount of evidence that the reorganization of the occipital cortex is functionally relevant and involved in the processing of language processing, Braille reading, spatial imagery and tactile discrimination [24], [30], [38], [39], [44], [61], [78], [79].

Based on the somatotopic reorganization found by Kupers et al. [44] we hypothesized that the reorganized occipital cortex of the blind might also be specifically suitable for processing language pertaining to space. Our results do not support this idea. The reorganized areas did not show a modulation for the spatial or relational conditions. Interestingly, we did find a significant difference between the blind and sighted individuals in the occipital cortex for the contrast *reversal > repetition*. The left middle occipital gyrus showed an increased activation for reversals in the blind. This might be related to the behavioral difference that blind participants responded slower to reversal trials than repetition trials, while this difference was absent in the sighted individuals. The reversal trials have a higher linguistic complexity since the relation between the persons was changed, but the situation described could still be the same (e.g. “Max left of Wies” and “Wies right of Max”). The activation in the left middle occipital gyrus could therefore be associated with processing linguistically more complex information in the blind. A negative correlation between the level of reorganization in the blind and their performance on linguistically more complex trials suggests this functional role. Participants with larger activity in the reorganized visual cortex responded more equally to reversal and repetition trials.

A possible explanation for the fact that the sighted participants responded equally fast to both reversal and repetition trials is that they used a visual-spatial strategy to solve the task. In contrast, the blind participants might rely more on a verbal strategy, which is more sensitive to linguistic complexity [62], [80]. If the blind were not relying on a spatial representation, but rather were solving the task using a propositional representation this would have resulted in distinct activation patterns for the blind and sighted. On the contrary, the large network of activation was very similar for both groups. The only significant differences were found in the occipital cortex, as explained above. Alternatively, the left SMG could be a linguistic node representing spatial prepositions, as such it would be sensitive to linguistic complexity. It should be noted here that no differences in activation between reversal and repetition trials were found within the left SMG. Thus, the linguistic complexity did not modulate the left SMG activation. Even with a possible difference in strategy between blind and sighted, both groups showed significant and comparable activation in the left SMG. This further strengthens the idea that a supramodal representation may underlie spatial language processing. Moreover, the concept of a supramodal representation does not exclude possible differences in strategy, such as previously found by Vanlierde et al. [24], [26] and described in the introduction. Rather it focuses on the common underlying types of information, such as spatial information elements.

In conclusion, the present study offers further insights in the patterns of brain activation underlying spatial language processing. We found that during language processing in general the blind and sighted individuals activate a highly similar network. In addition the blind participants also showed activation in the reorganized occipital cortex. This reorganization activity was also found in the spatial language task, and was modulated by linguistic complexity. There thus seems to be a functional relevance of the reorganized areas; they support processing of linguistically more complex trials. However, within these regions there is no further distinction for a particular semantic category, in this case spatial relations. Importantly, the left SMG appears particularly involved in parsing relational terms ordered along a single dimension. The finding that blind participants also activate the left SMG when processing relational terms is very interesting since it implies that the role of the SMG is hard-wired. Regardless of visual experience, the left SMG supports the supramodal representation of spatial and other dimensional relations in language.

## References

[1] Amorapanth PX, Widick P, Chatterjee A (2010). The neural basis for spatial relations.. Journal of Cognitive Neuroscience.

[2] Kemmerer D (2005). The spatial and temporal meanings of English prepositions can be independently impaired.. Neuropsychologia.

[3] Tranel D, Kemmerer D (2004). Neuroanatomical correlates of locative prepositions.. Cognitive Neuropsychology.

[4] Noordzij ML, Neggers SFW, Ramsey NF, Postma A (2008). Neural correlates of locative prepositions.. Neuropsychologia.

[5] Damasio H, Grabowski TJ, Tranel D, Ponto LL, Hichwa RD (2001). Neural correlates of naming actions and of naming spatial relations.. NeuroImage.

[6] Emmorey K, Damasio H, McCullough S, Grabowski TJ, Ponto LLB (2002). Neural systems underlying spatial language in american sign language.. NeuroImage.

[7] Barsalou LW (1999). Perceptual symbol systems.. Behavioral and Brain Sciences.

[8] Bonino D, Ricciardi E, Sani L, Gentili C, Vanello N (2008). Tactile spatial working memory activates the dorsal extrastriate cortical pathway in congenitally blind individuals.. Archives Italiennes de Biologie.

[9] Pietrini P, Furey ML, Ricciardi E, Gobbini MI, Wu WHC (2004). Beyond sensory images: Object-based representation in the human ventral pathway.. Proceedings of the National Academy of Sciences of the United States of America.

[10] Ricciardi E, Bonino D, Gentili C, Sani L, Pietrini P (2006). Neural correlates of spatial working memory in humans: A functional magnetic resonance imaging study comparing visual and tactile processes.. Neuroscience.

[11] Struiksma ME, Noordzij ML, Postma A (2009). What is the link between language and spatial images? Behavioral and neural findings in the blind and sighted.. Acta Psychologica.

[12] Cattaneo Z, Vecchi T (2011). Blind Vision: The Neuroscience of Visual Impairment..

[13] Thinus-Blanc C, Gaunet F (1997). Representation of space in blind persons: Vision as a spatial sense?. Psychological Bulletin.

[14] Knauff M, May E (2006). Mental imagery, reasoning, and blindness.. Quarterly Journal of Experimental Psychology.

[15] Jackendoff R, Bloom P, Peterson MA, Nadel L, Garrett MF (1996). The architecture of the linguistic-spatial interface.. Language and space, Language, speech, and communication.

[16] Farah MJ, Hammond KM, Levine DN, Calvanio R (1988). Visual and spatial mental imagery: dissociable systems of representation.. Cognitive Psychology.

[17] Aleman A, Van Lee L, Mantione MH, Verkoijen IG, De Haan EHF (2001). Visual imagery without visual experience: evidence from congenitally totally blind people.. NeuroReport.

[18] Kerr NH (1983). The role of vision in “visual imagery” experiments: evidence from the congenitally blind.. Journal of Experimental Psychology: General.

[19] Klatzky RL, Golledge RG (1995). Performance of blind and sighted persons on spatial tasks.. Journal of Visual Impairment & Blindness.

[20] Zimler J, Keenan JM (1983). Imagery in the congenitally blind: How visual are visual images?. Journal of Experimental Psychology: Learning, Memory, and Cognition.

[21] Cattaneo Z, Vecchi T, Cornoldi C, Mammarella I, Bonino D (2008). Imagery and spatial processes in blindness and visual impairment.. Neuroscience & Biobehavioral Reviews.

[22] Vecchi T, Tinti C, Cornoldi C (2004). Spatial memory and integration processes in congenital blindness.. NeuroReport.

[23] Stilla R, Hanna R, Hu X, Mariola E, Deshpande G (2008). Neural processing underlying tactile microspatial discrimination in the blind: A functional magnetic resonance imaging study.. Journal of Vision.

[24] Vanlierde A, De Volder AG, Wanet-Defalque M-C, Veraart C (2003). Occipito-parietal cortex activation during visuo-spatial imagery in early blind humans.. NeuroImage.

[25] Mahon BZ, Anzellotti S, Schwarzbach J, Zampini M, Caramazza A (2009). Category-specific organization in the human brain does not require visual experience.. Neuron.

[26] Vanlierde A, Wanet-Defalque M-C (2004). Abilities and strategies of blind and sighted subjects in visuo-spatial imagery.. Acta Psychologica.

[27] Lacey S, Campbell C, Sathian K (2007). Vision and touch: Multiple or multisensory representations of objects?. Perception.

[28] Noppeney U (2007). The effects of visual deprivation on functional and structural organization of the human brain.. Neuroscience and Biobehavioral Reviews.

[29] Noppeney U, Friston KJ, Ashburner J, Frackowiak RSJ, Price CJ (2005). Early visual deprivation induces structural plasticity in gray and white matter.. Current Biology.

[30] Pascual-Leone A, Amedi A, Fregni F, Merabet LB (2005). The plastic human brain cortex.. Annual Review of Neuroscience.

[31] Shimony JS, Burton H, Epstein AA, McLaren DG, Sun SW (2006). Diffusion tensor imaging reveals white matter reorganization in early blind humans.. Cerebral Cortex.

[32] Amedi A, Floel A, Knecht S, Zohary E, Cohen LG (2004). Transcranial magnetic stimulation of the occipital pole interferes with verbal processing in blind subjects.. Nature Neuroscience.

[33] Burton H, Snyder AZ, Conturo TE, Akbudak E, Ollinger JM (2002). Adaptive changes in early and late blind: a fMRI study of Braille reading.. Journal of Neurophysiology.

[34] Gizewski ER, Gasser T, de Greiff A, Boehm A, Forsting M (2003). Cross-modal plasticity for sensory and motor activation patterns in blind subjects.. NeuroImage.

[35] Melzer P, Morgan VL, Pickens DR, Price RR, Wall RS (2001). Cortical activation during Braille reading is influenced by early visual experience in subjects with severe visual disability: A correlational fMRI study.. Human Brain Mapping.

[36] Sadato N, Pascual-Leone A, Grafmani J, Ibanez V, Deiber MP (1996). Activation of the primary visual cortex by Braille reading in blind subjects.. Nature.

[37] Sathian K (2005). Visual cortical activity during tactile perception in the sighted and the visually deprived.. Developmental Psychobiology.

[38] Uhl F, Franzen P, Lindinger G, Lang W, Deecke L (1991). On the functionality of the visually deprived occipital cortex in early blind persons.. Neuroscience Letters.

[39] Cohen LG, Celnik P, Pascual-Leone A, Corwell B, Faiz L (1997). Functional relevance of cross-modal plasticity in blind humans.. Nature.

[40] Raz N, Amedi A, Zohary E (2005). V1 activation in congenitally blind humans is associated with episodic retrieval.. Cerebral Cortex.

[41] Burton H, Snyder AZ, Diamond JB, Raichle ME (2002). Adaptive changes in early and late blind: a fMRI study of verb generation to heard nouns.. Journal of Neurophysiology.

[42] Matteau I, Kupers R, Ricciardi E, Pietrini P, Ptito M (2010). Beyond visual, aural and haptic movement perception: hMT+ is activated by electrotactile motion stimulation of the tongue in sighted and in congenitally blind individuals.. Brain Research Bulletin.

[43] Ptito M, Kupers R (2005). Cross-modal plasticity in early blindness.. Journal of Integrative Neuroscience.

[44] Kupers R, Fumal A, de Noordhout AM, Gjedde A, Schoenen J (2006). Transcranial magnetic stimulation of the visual cortex induces somatotopically organized qualia in blind subjects.. Proceedings of the National Academy of Sciences.

[45] Hamilton R, Keenan JP, Catala M, Pascual-Leone A (2000). Alexia for Braille following bilateral occipital stroke in an early blind woman.. NeuroReport.

[46] Merabet LB, Thut G, Murray B, Andrews J, Hsiao S (2004). Feeling by sight or seeing by touch?. Neuron.

[47] Logan GD, Compton BJ (1996). Distance and distraction effects in the apprehension of spatial relations.. Journal of Experimental Psychology: Human Perception and Performance.

[48] Amedi A, Raz N, Pianka P, Malach R, Zohary E (2003). Early ‘visual’ cortex activation correlates with superior verbal memory performance in the blind.. Nature Neuroscience.

[49] Binder JR, Frost JA, Hammeke TA, Bellgowan PS, Springer JA (2000). Human temporal lobe activation by speech and nonspeech sounds.. Cerebral Cortex.

[50] Neggers SFW, Hermans E, Ramsey NF (2008). Enhanced sensitivity with fast three-dimensional blood-oxygen-level-dependent functional MRI: comparison of SENSE-PRESTO and 2D-EPI at 3 T.. NMR in Biomedicine.

[51] Klarhöfer M, Dilharreguy B, van Gelderen P, Moonen CT (2003). A PRESTO-SENSE sequence with alternating partial-Fourier encoding for rapid susceptibility-weighted 3D MRI time series.. Magnetic Resonance in Medicine.

[52] Ashburner J, Friston KJ (2005). Unified segmentation.. NeuroImage.

[53] Crinion J, Ashburner J, Leff A, Brett M, Price CJ (2007). Spatial normalization of lesioned brains: Performance evaluation and impact on fMRI analyses.. NeuroImage.

[54] Coventry KR, Garrod SC (2004). Saying, seeing and acting.. The psychological semantics of spatial prepositions.

[55] Friston KJ, Penny WD, Glaser DE (2005). Conjunction revisited.. NeuroImage.

[56] Nichols T, Brett M, Andersson J, Wager T, Poline J-B (2005). Valid conjunction inference with the minimum statistic.. NeuroImage.

[57] Friston KJ, Holmes A, Poline JB, Price CJ, Frith CD (1996). Detecting activations in PET and fMRI: levels of inference and power.. NeuroImage.

[58] Friston KJ (1997). Testing for anatomically specified regional effects.. Human Brain Mapping.

[59] Ackermann H, Wildgruber D, Daum I, Grodd W (1998). Does the cerebellum contribute to cognitive aspects of speech production? A functional magnetic resonance imaging (fMRI) study in humans.. Neuroscience Letters.

[60] Herholz K, Thiel A, Wienhard K, Pietrzyk U, von Stockhausen HM (1996). Individual functional anatomy of verb generation.. NeuroImage.

[61] Van der Lubbe RHJ, Van Mierlo CM, Postma A (2010). The involvement of occipital cortex in the early blind in auditory and tactile duration discrimination tasks.. Journal of Cognitive Neuroscience.

[62] Noordzij ML, Van der Lubbe RHJ, Postma A (2006). Electrophysiological support for strategic processing of spatial sentences.. Psychophysiology.

[63] Ricciardi E, Bonino D, Sani L, Pietrini P, Bicchi A, Buss M, Ernst MO, Peer A (2008). Functional exploration studies of supramodal organization in the human extrastriate cortex.. The Sense of Touch and its Rendering.

[64] Poirier C, Collignon O, Scheiber C, Renier L, Vanlierde A (2006). Auditory motion perception activates visual motion areas in early blind subjects.. NeuroImage.

[65] Ricciardi E, Vanello N, Sani L, Gentili C, Scilingo EP (2007). The effect of visual experience on the development of functional architecture in hMT+.. Cerebral Cortex.

[66] Ricciardi E, Bonino D, Sani L, Vecchi T, Guazzelli M (2009). Do we really need vision? How blind people “see” the actions of others.. Journal of Neuroscience.

[67] Von Senden M (1932). Raum und Gestalt: Auffassung bei operierten Blindgeborenen vor und nach del Operation..

[68] Pascual-Leone A, Hamilton R (2001). The metamodal organization of the brain.. Progress in Brain Research.

[69] Held R, Ostrovsky Y, de Gelder B, Gandhi T, Ganesh S (2011). The newly sighted fail to match seen with felt.. Nature Neuroscience.

[70] Boroditsky L (2000). Metaphoric structuring: understanding time through spatial metaphors.. Cognition.

[71] Haspelmath M (1997). From space to time: temporal adverbials in the world's languages..

[72] Heine B, Claudi U, Hünnemeyer F (1991). Grammaticalization..

[73] Santiago J, Lupiáñez J, Pérez E, Funes MJ (2007). Time (also) flies from left to right.. Psychonomic Bulletin & Review.

[74] Kriegeskorte N, Simmons WK, Bellgowan PSF, Baker CI (2009). Circular analysis in systems neuroscience: the dangers of double dipping.. Nature Neuroscience.

[75] Bookheimer SY (2002). Functional MRI of language: new approaches to understanding the cortical organization of semantic processing.. Annual Review of Neuroscience.

[76] Cuenod CA, Bookheimer SY, Hertz-Pannier L, Zeffiro TA, Theodore WH (1995). Functional MRI during word generation, using conventional equipment: A potential tool for language localization in the clinical environment.. Neurology.

[77] Gernsbacher MA, Kaschak MP (2003). Neuroimaging studies of language production and comprehension.. Annual Review of Psychology.

[78] Ofan RH, Zohary E (2007). Visual cortex activation in bilingual blind individuals during use of native and second language.. Cerebral Cortex.

[79] Röder B, Stock O, Bien S, Neville H, Rösler F (2002). Speech processing activates visual cortex in congenitally blind humans.. European Journal of Neuroscience.

[80] Noordzij ML, Van der Lubbe RHJ, Postma A (2005). Strategic and automatic components in the processing of linguistic spatial relations.. Acta Psychologica.

